# IL-21 isoform transgenic mice spontaneously develop mammary tumors: possible involvement of IL-21-induced osteopontin expression in tumorigenesis

**DOI:** 10.3389/fimmu.2026.1834095

**Published:** 2026-06-01

**Authors:** Risako Yamaguchi, Akemi Araki, Yuji Takeda, Shinichi Saitoh, Junji Yokozawa, Masahiro Yamamoto, Naing Ye Aung, Mitsuru Futakuchi, Satoru Nagase, Hironobu Asao

**Affiliations:** 1 Department of Immunology, Yamagata University Faculty of Medicine, Yamagata, Japan; 2 Department of Obstetrics and Gynecology, Yamagata University Faculty of Medicine, Yamagata, Japan; 3 Department of Tumor Pathology, Faculty of Life Sciences, Kumamoto University, Kumamoto, Japan; 4 Department of Pathology, Yamagata University Faculty of Medicine, Yamagata, Japan

**Keywords:** immunosuppression, interleukin-21, interleukin-21 isoform, mammary tumor, osteopontin

## Abstract

**Introduction:**

Interleukin-21 (IL-21) is a pleiotropic cytokine produced by CD4^+^ T cells that critically regulates the differentiation and functions of various adaptive and innate immune cells. Emerging evidence suggests that IL-21 production increases in CD4^+^ T cells during aging. However, the long-term impact of sustained IL-21 exposure on peripheral tissues remains unclear.

**Methods:**

To address this question, we generated transgenic mice expressing a membrane-bound IL-21 isoform specifically in T cells (IL-21isoTg mice) and analyzed age-associated tissue alterations.

**Results:**

Female IL-21isoTg mice developed progressive mammary gland dysplasia beginning at approximately 14 weeks of age, and more than 45% developed mammary tumors after 12 months. Comprehensive gene expression analysis of mammary epithelial cells (MECs) isolated prior to tumor onset (10 weeks of age) revealed robust upregulation of the *Spp1* gene encoding osteopontin (OPN) in IL-21isoTg mice. Quantitative RT-PCR confirmed a significant increase in *Spp1* expression in both MECs and mammary stromal cells of IL-21isoTg mice. Given the established role of OPN in shaping the tumor microenvironment (TME) and promoting immunosuppression, we investigated the mechanism underlying *Spp1* induction. Using splenocytes as a surrogate model for mammary stromal immune cells, we found that IL-21 stimulation selectively enhanced OPN expression in macrophages and B cells. In contrast, cytokines previously reported to induce *Spp1*, including IL-1β, IL-6, IL-17A, and TNF-α, failed to significantly upregulate *Spp1* in this context, indicating an IL-21-specific effect. Although IL-21 did not directly affect mammary epithelial tumor cell lines, it induced splenocyte-derived IL-6 and TNF-α, which subsequently promoted *Spp1* expression in tumor cell lines. These findings suggest that in IL-21isoTg mice, T cells accumulating in the mammary gland produce IL-21iso, which directly induces OPN expression in macrophages and B cells, and indirectly enhances OPN expression in MECs through IL-6 and TNF-α. Age-associated increases in IL-21 production may, therefore, promote mammary tumor development and progression by enhancing OPN expression, leading to local inflammation and immunosuppression within the mammary tissue.

## 1 Introduction

Interleukin-21 (IL-21) and its receptor (IL-21R) were identified as members of the cytokine and cytokine receptor families (1, 2). IL-21R forms a heterodimeric complex with the common γ-chain and transduces signals predominantly through activation of STAT3 upon ligand engagement (3, 4). IL-21, mainly produced by activated CD4^+^ and natural killer T cells (1), exerts pleiotropic immunoregulatory functions (5). Among the CD4^+^ T cell subsets, T helper 17 (Th17) cells are a major source of IL-21, which contributes to the differentiation and function of the Th17 lineage in an autocrine manner (6–12). In addition, follicular helper T cells produce IL-21 and, together with IL-4 and CD40L, regulate B cell activation and antibody production (13–15).

IL-21 also cooperates with other γc cytokines, such as IL-7 and IL-15, to regulate CD8^+^ T cell proliferation, activation, and memory formation (16–20). Notably, IL-21 sustains the function of exhausted CD8^+^ T cells during chronic viral infections and limits T cell exhaustion, thereby contributing to viral control (21–25). Collectively, these findings establish IL-21 as a central modulator of adaptive immunity.

To elucidate the *in vivo* functions of IL-21, we initially attempted to generate IL-21 transgenic mice. However, stable lines could not be established. Therefore, we generated transgenic mice expressing a membrane-bound IL-21 isoform (IL-21iso) that retains functional properties of secreted IL-21 (26, 27) specifically in T cells (IL-21isoTg mice). IL-21isoTg mice were born according to Mendelian ratios and exhibited no prominent phenotypic differences from wild-type mice at birth. However, DSS-induced colitis was significantly exacerbated in IL-21isoTg mice compared to wild-type mice, with a marked increase in inflammatory responses (28).

In humans, CD4^+^ T cells from elderly individuals produce higher levels of IL-21 than those from younger individuals, and this increase correlates with cytomegalovirus infection (29). Elevated circulating IL-21 levels have also been reported in elderly patients with Alzheimer’s disease or mild cognitive impairment, suggesting systemic consequences of chronic IL-21 exposure (30, 31). These observations suggest that sustained IL-21 production during aging influences peripheral tissue homeostasis and disease susceptibility. Therefore, to investigate the effects of long-term IL-21 exposure, we aged IL-21isoTg mice and evaluated their natural course.

Osteopontin (OPN) is a multifunctional secreted phosphoprotein initially identified in cancer cells (32); however, its pivotal roles in bone remodeling, angiogenesis, and immunomodulation are now well-established (33). Beyond its physiological functions, OPN contributes to tumor progression, metastasis, and immunosuppression across various human malignancies (34–36). Elevated OPN expression has been reported in multiple cancers, including breast cancer, and correlates with poor prognosis (37, 38). Recent studies have also demonstrated that OPN derived from tumor-associated macrophages promotes CD8^+^ T cell exhaustion (39–41).

In this study, we identified robust OPN expression in the mammary epithelium and stromal cells of IL-21isoTg mice. Given its significant role in tumor-associated immunomodulation, we investigated the involvement of OPN in mammary tumor development in this model.

## 2 Materials and methods

### 2.1 Mice

**Figure 1:**
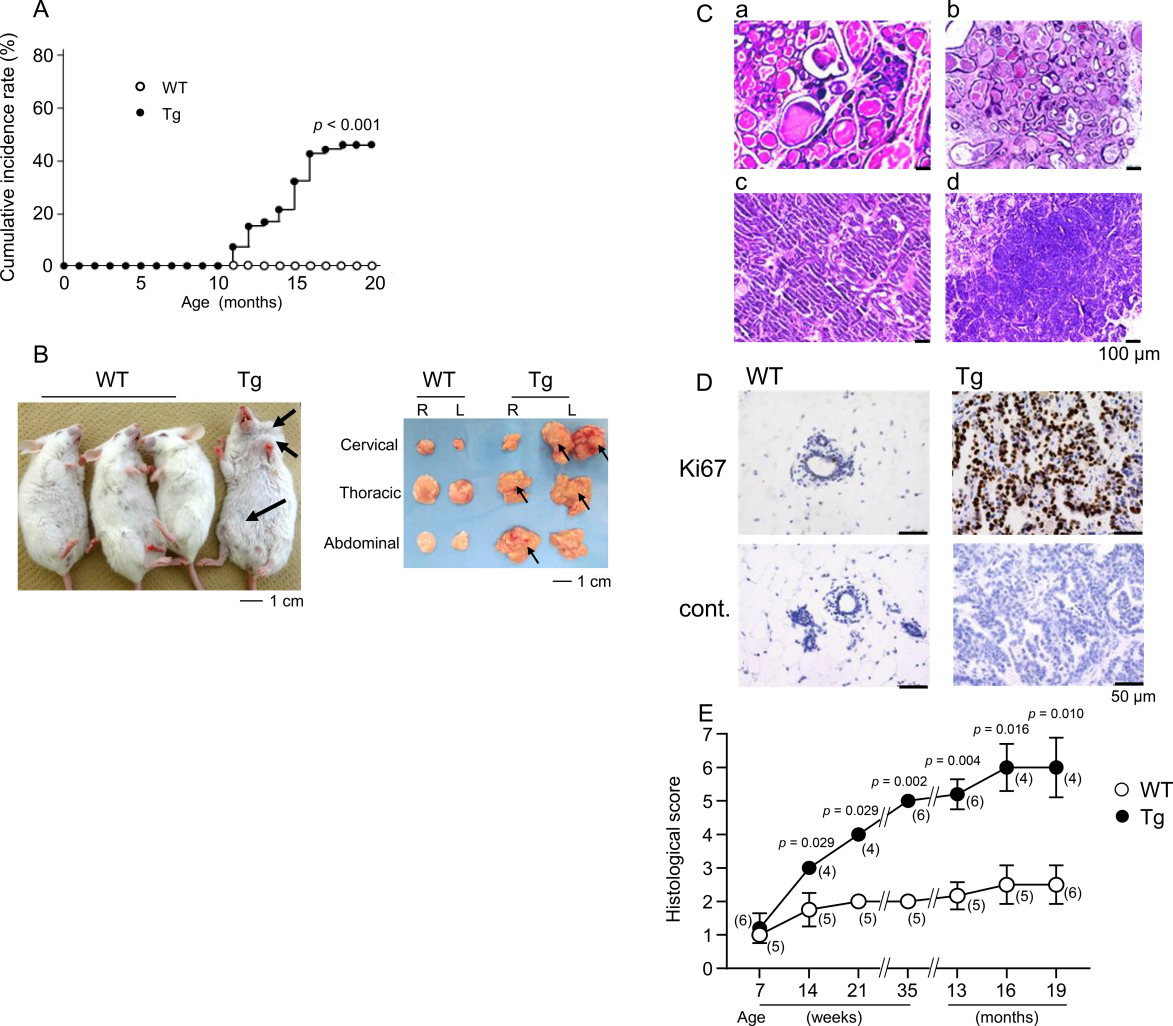
Spontaneous development of mammary tumors in female IL-21isoTg mice. **(A)** Cumulative incidence (%) of mammary tumors in wild-type (open circles, n = 65) and IL-21isoTg mice (closed circles, n = 65). Statistical significance of difference was evaluated using the log-rank test. **(B)** Representative gross appearance of wild-type and IL-21isoTg mice (left) and macroscopic view of mammary glands (right). Arrows indicate tumor sites. R, right; L, left. **(C)** Hematoxylin and eosin staining of mammary tumors from 11–17-month-old IL-21isoTg mice. **(a, b)** Tubular adenoma. **(c, d)** Adenocarcinoma. **(D)** Ki67 immunostaining of mammary tissue from a 15-month-old wild-type mouse (left) and mammary tumor from a 15-month-old IL-21isoTg mouse (right) (upper panels). Isotype control staining is shown in the lower panels. **(E)** Histological scoring of age-related changes in mammary gland tissues from IL-21isoTg and wild-type mice without macroscopically detectable tumors. The number of mice in each group is indicated in parentheses in the figure. Statistical analysis was performed using the Mann–Whitney U test.

IL-21isoTg mice on the BALB/c background were generated as previously described (28). Mice were maintained under specific pathogen-free conditions with a 12-h light/dark cycle and provided *ad libitum* access to standard chow and water until the designated age. For the long-term observation cohort shown in **Figure 1A**, a total of 65 animals per group were analyzed. This represents the cumulative total from multiple experimental cohorts monitored over a 5-year period. Each cohort consisted of 2–4 female wild-type and transgenic littermates, which were followed for up to 20 months. For other experiments, independent mice separate from this long-term cohort were used, and the results represent the summary of at least three independent experimental replicates.

All female mice used for mammary tissue analysis were virgin, and their estrous cycle stages were randomized to minimize potential hormonal influence. Eight-week-old Tg mice were randomly assigned to the ovariectomy group (n = 9) or the sham-operated group (n = 7). For anesthesia, a mixture of medetomidine (0.3 mg/kg), midazolam (4 mg/kg), and butorphanol (5 mg/kg) was administered subcutaneously. These mice were observed for a period of up to approximately 20 months. All animal experiments were conducted in accordance with the guidelines of the Laboratory Animal Center of the Yamagata University Faculty of Medicine and approved by the Institutional Animal Care and Use Committee of the Yamagata University Faculty of Medicine (approval no. R7083).

### 2.2 Histopathological analysis

After euthanasia, the entire mammary glands from both the left and right sides were collected and analyzed individually. Mammary tissues were fixed with 4% paraformaldehyde in phosphate-buffered saline, embedded in paraffin, and sectioned. The sections were stained with hematoxylin and eosin according to standard procedures.

For immunohistochemical studies, monoclonal antibodies against Ki67 (clone sp6; ab16667, Abcam, Cambridge, UK) and Osteopontin (clone EPR21138; ab218237, Abcam) were used. Tissue samples were collected from the fourth inguinal mammary gland. All procedures were carried out using the BOND RXm autostainer (Leica Biosystems, Nussloch, Germany) according to the manufacturer’s protocols. Immunoreactivity was visualized using brown staining with 3,3’-diaminobenzidine (DAB; BOND Polymer Refine Detection, Leica Biosystems), and sections were counterstained with hematoxylin (BOND Polymer Refine Detection, Leica Biosystems).

### 2.3 Histological scoring of mammary tissue

Hematoxylin and eosin-stained mammary gland sections were examined microscopically and scored as follows:

1 point, few normal ducts;
2 points, numerous normal ducts;
3 points, few dilated ducts;
4 points, numerous dilated ducts;
5 points, markedly increased dilated ducts;
6 points, tubular adenoma;
7 points, adenocarcinoma

The highest score among all mammary gland tissues in each mouse was taken as the score for that mouse.

### 2.4 Whole-mount staining of mammary glands

The fourth inguinal mammary gland was dissected and spread on a glass slide. Tissues were fixed overnight at 4 °C in 4% paraformaldehyde and stained overnight at 20˚C with 0.2% carmine and 0.5% aluminum potassium sulfate, as previously described (42, 43).

### 2.5 Isolation and purification of MECs

MECs were isolated using previously described methods with minor modifications (44, 45). Mammary tissue was minced into approximately 1 mm² fragments and digested in RPMI-1640 containing 5% fetal calf serum, 1 mg/mL collagenase type IV (Sigma), and 0.05 mg/mL DNase I (Sigma) at 37 °C for 90 min with gentle shaking (95 rpm).

After digestion, the cells were washed twice with phosphate-buffered saline and treated with the ACK buffer to remove erythrocytes. Cells were further incubated with 0.125% trypsin at 37 °C for 2 min, followed by treatment with 5 mg/mL dispase and 0.1 mg/mL DNase I in RPMI-1640 with 5% fetal calf serum at 37 °C for 2 min. The cell suspension was filtered through a 40 μm mesh.

Negative selection was performed using PE-conjugated antibodies against CD45, CD31, and CD140a. The cells were then incubated with BD IMag anti-PE magnetic particles (BD Biosciences), according to the manufacturer’s protocol.

### 2.6 RNA isolation and quantitative real-time RT-PCR

Total RNA was extracted using TRIzol™ Reagent (Invitrogen). cDNA was synthesized from 0.1 μg total RNA using the ReverTra Ace™ qPCR RT Master Mix with gDNA Remover (TOYOBO) according to the manufacturer’s instructions.

Quantitative PCR was performed using TB Green Premix Ex Taq™ II FAST (TaKaRa Bio) on a CFX96 Real-Time PCR Detection System (Bio-Rad). Relative gene expression was calculated using the 2^−ΔΔCt^ method, with mouse Actb mRNA expression levels as internal control. The primer sequences are listed in **Table 1**.

**Table 1:** Primers used.

Gene	Forward primer (5’→3’)	Reverse primer (5’→3’)
*Actb*	TGACAGGATGCAGAAGGAGA	GCTGGAAGGTGGACAGTGAG
*Epcam*	TTGCAGACTGCGCTTCAAGA	ACTCGGGTGCCTTTTCATCA
*Erbb2*	GGTGTGAAGCCAGACCTCTC	GGATCTTCTGTCGCCTTCGT
*Esr1*	CTGCCAAGGAGACTCGCTAC	TCTTTCCGTATGCCGCCTTT
*Il1b*	GTTGACGGACCCCAAAAGATG	CCTCATCCTGGAAGGTCCAC
*Il6*	TGGAGTACCATAGCTACCTGGA	TGACTCCAGCTTATCTGTTAGGAG
*Il10*	CCCTGGGTGAGAAGCTGAAGAC	ACCTGCTCCACTGCCTTGC
*Il17a*	CTGAGGCCAAGGACTTCCTCCA	GGCACTGAGCTTCCCAGATCAC
*Il21*	TGGCTCCTTCAAAAGATGAT	AAACTAGTATGTACTCCTGCATTCGTG
*Il21iso*	GAGGAAAGAAACAGAAGCACA	AGACACAACATGGAAGTGAAA
*Krt7*	AAGGGGAGCTGGCAATCAAG	CCCATGGTTCCTCCGAAGAT
*Pgr*	GATGTGGTCTATGCAGGGCA	ATGCTTGTACGACCTCCACC
*Prlr*	TGCACTTGCTTACATGCTGC	TTGGGGCCACTGGTTTTGTA
*Spp1*	TGGTGGTGATCTAGTGGTG	CATGGTCGTAGTTAGTCCTG
*Tnf*	ACCCTCACACTCAGATCATC	GAGTAGACAAGGTACAACCC

### 2.7 Bulk RNA sequencing

Total RNA from MECs was extracted using TRIzol™ Reagent and treated with DNase I. RNA quality was assessed using a 4200 TapeStation system (Agilent). Poly(A)-selected RNA libraries from IL-21isoTg and wild-type mice (n = 3 per group) were sequenced using the Illumina NovaSeq 6000 platform with 150-bp paired-end reads (Rhelixa, Tokyo, Japan). Raw sequencing data were evaluated using FastQC (v0.11.7).

### 2.8 Flow cytometry

Isolated mammary gland cells, splenocytes, 5T7 cells, and 2T10 cells were analyzed using flow cytometry. For cell-surface antigen analysis, cells were stained with fluorochrome-conjugated monoclonal antibodies (mAbs). For intracellular staining of OPN and IL-21, cells were fixed and permeabilized using Fixation and Permeabilization Solution (BD Biosciences, #554722), followed by staining with labeled antibodies in Intracellular Staining Wash Buffer (BioLegend, #421002) in the presence of an Fc-blocking antibody (anti-CD16/32, clone 2.4G2). Ki67 staining was performed using 70% ethanol according to the BioLegend protocol. Peripheral blood was collected via retro-orbital bleeding, and after red blood cell lysis with ACK buffer, lymphocytes were separated. Foxp3 staining was performed using the eBioscience™ Foxp3/Transcription Factor Staining Buffer Set (#00-5523). Dead cells were excluded using Zombie NIR (BioLegend). Data were acquired using a FACSMelody™ flow cytometer (BD Biosciences) and analyzed using FlowJo v10 software.

### 2.9 Antibodies and cytokines

Antibodies used in this study were PE-anti-CD45, APC-anti-CD45 and FITC-anti-CD45 (clone 30-F11, BioLegend), PE-anti-CD31 and APC-anti-CD31 (clone 390, BioLegend), PE-anti-CD140a and APC-anti-CD140a (clone APA5, BioLegend), Pacific Blue-anti-CD24 (clone M1/69, BioLegend), FITC-anti-CD49f (clone GoH3, BioLegend), APC-anti-F4/80 and PE-anti-F4/80 (clone BM8, BioLegend), Pacific Blue-anti-CD11b (clone M1/70, BioLegend), PE-anti-CD206 (clone C068C2, BioLegend), FITC-anti-CD86 (clone PO3, BioLegend), APC-anti-I-A/I-E and APC/Fire™-anti-I-A/I-E (clone M5/114.15.2, BioLegend), FITC-anti-CD3 (clone 17A2, BioLegend), FITC-anti-CD3ϵ (clone 145-2C11, Biolegend), Pacific Blue-anti-CD3 (clone 17A2, BioLegend), Pacific Blue-anti CD4 (clone GK1.5, Biolegend), PerCP/Cyanine5.5-anti-CD11c (clone N418, BioLegend), PE-anti-CD45R/B220 (clone RA3-6B2, BioLegend), PerCP/Cyanine5.5-anti-Ly-6C (clone HK1.4, BioLegend), Brilliant Violet 421™-anti-Ly-6G (clone 1A8, BioLegend), Brilliant Violet 421™-anti-Ly-6G/Ly-6C (Gr1) (clone RB6-8C5, BioLegend), CoraLite Plus 647-anti-osteopontin (Proteintech), CoraLite Plus 647-IgG isotype control (Proteintech), PE-anti-Ki67 (clone 11F6, BioLegend), PE-anti-Foxp3 (clone FJK-16s, eBioscience), anti-Ki67 (clone sp6, abcam), anti-Osteopontin (clone EPR21138, Abcam) and APC-anti-IL-21 (clone S200178, BioLegend).

Recombinant cytokines used included mouse IL-1 beta (R&D systems), human IL-6 (Peprotech), mouse IL-17A (R&D systems), mouse IL-21 (R&D systems), human TNF-α (R&D systems).

### 2.10 Cell lines and culture

The 5T7 and 2T10 cell lines were derived from spontaneous thoracic mammary tumors identified in IL-21isoTg mice at 12 and 15 months of age, respectively. To stabilize their tumorigenic properties, each tumor was initially transplanted subcutaneously into syngeneic mice and serially passaged twice *in vivo*. Subsequently, the resulting tumor tissues were harvested, dissociated, and subjected to continuous *in vitro* culture to establish the stable cell lines. Murine splenocytes were prepared by mechanical dissociation and erythrocyte lysis in the ACK buffer.

Cells were cultured in RPMI 1640 supplemented with 10% fetal calf serum, 50 μM 2-mercaptoethanol, 100 U/mL penicillin, and 10 μg/mL streptomycin. Renca cells (CRL-2947) were provided by Professor Norihiko Tsuchiya (Yamagata University, Japan).

### 2.11 Tumor transplantation

Seventeen-to-eighteen-week-old mice were injected subcutaneously with 5 × 10^5^ Renca cells into the right dorsal flank. Tumor size was measured using digital calipers, and tumor volume was calculated as follows:

(tumor volume; mm^3^) = (major axis; mm) x (minor axis; mm)^2^ x 0.5236 (46).

### 2.12 ELISA

Serum estradiol and prolactin levels were measured using ELISA kits for estradiol (Calbiotech) and mouse prolactin (Abcam), respectively, according to the manufacturers’ protocols.

### 2.13 Statistical analysis

Data are presented as the mean ± standard deviation. Data from independent experiments were pooled as appropriate. Statistical tests are specified in the respective Figure legends. Statistical analyses were performed using GraphPad Prism version 8. Differences were considered statistically significant if the null hypothesis could be rejected at the 0.05 probability level.

## 3 Results

### 3.1 Female IL-21isoTg mice develop mammary tumors at a high frequency

When IL-21isoTg mice were maintained under specific pathogen-free conditions, palpable mammary tumors became apparent from approximately 12 months of age. The incidence rate exceeded 45% at 20 months of age (**Figure 1A**). In contrast, no mammary tumors were observed in the wild-type mice. Images of 16-month-old wild-type mice and tumor-bearing IL-21isoTg mice, including photos of mammary tissues, are shown in **Figure 1B**. Tumors developed in multiple mammary glands, particularly in the thoracic mammary glands (**Supplementary Figure 1A**).

Hematoxylin and eosin-stained sections of mammary tumors from IL-21isoTg mice aged 11–17 months are shown in **Figure 1C**. Histopathological examination revealed features consistent with tubular adenoma (a, b) and adenocarcinoma (c, d). To evaluate the proliferative index of tumor cells, adenocarcinoma tissues were stained for Ki67. Ki67 expression was clearly detected in tumor tissues from IL-21isoTg mice but not in samples from age-matched wild-type littermates (**Figure 1D**).

Furthermore, Ki67 staining of mammary tissues from IL-21isoTg mice without overt tumor formation demonstrated a significantly increased number of Ki67-positive cells compared with that in wild-type mice (**Supplementary Figure 1B**).

Whole-mount staining of mammary glands from 20-week-old mice revealed that IL-21isoTg mice exhibited marked mammary gland development and ductal dilation compared to their wild-type counterparts (**Supplementary Figure 1C**).

To further assess early histological changes, mice without macroscopically detectable tumors were sacrificed, and mammary gland alterations were scored. Ductal dilation was observed in IL-21isoTg mice as early as at 14 weeks of age and its severity progressively increased with age. With advancing age, a substantial proportion of IL-21isoTg mice developed tubular adenomas and adenocarcinomas (**Figure 1E**).

### 3.2 Increased numbers of MECs and M2 macrophages in IL-21isoTg mice prior to tumor development

**Figure 2:**
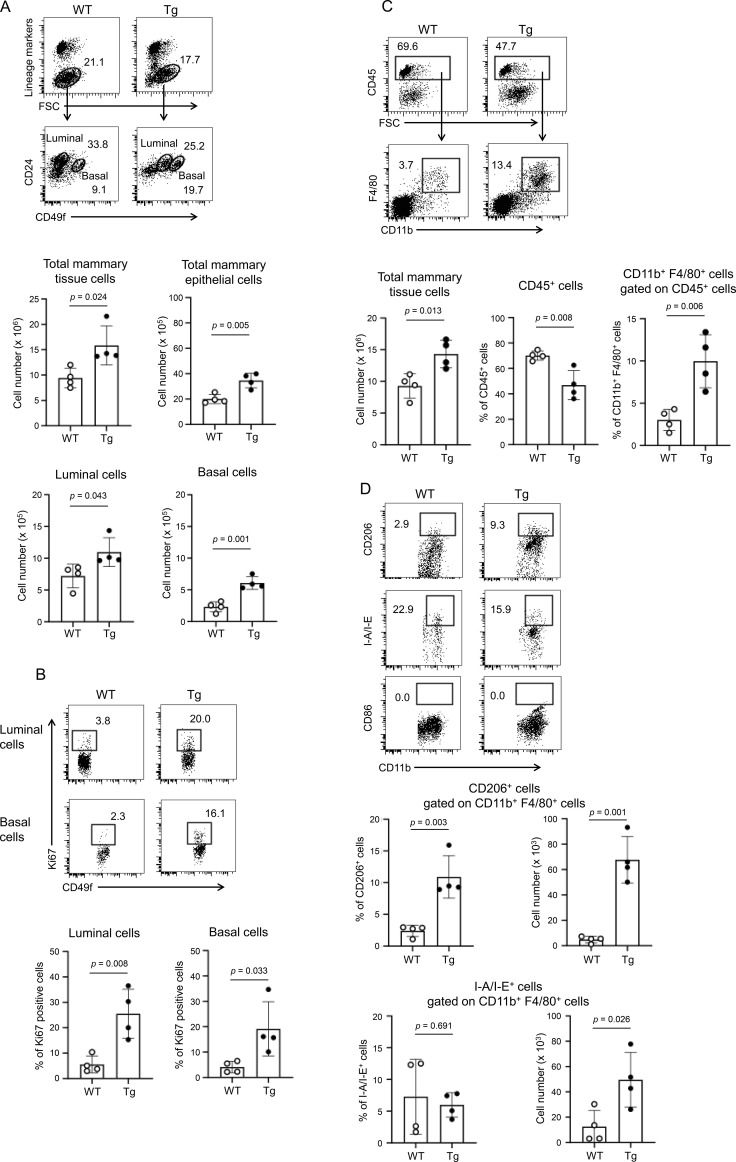
Increased abundance of MECs and M2 macrophages in IL-21isoTg mice. **(A)** Flow cytometry analysis of MECs from 16–19-week-old mice. Antibodies against lineage markers CD45, CD31, and CD140a were used to exclude hematopoietic, endothelial, and fibroblast cells. Lineage-negative cells were gated and analyzed for CD24 and CD49f expression (upper panels). Total mammary cells and their subsets (mammary, luminal, and basal cells) are shown (lower panels). n = 4. Mann–Whitney U test. **(B)** Flow cytometry analysis of Ki67-positive luminal and basal cells from 16–19-week-old mice (upper panels). Frequencies of Ki67-positive cells are shown (lower panels). n = 4. Mann–Whitney U test. **(C)** Flow cytometry analysis of CD11b^+^F4/80^+^ macrophages among CD45^+^ cells accumulated in mammary tissues from 30–35-week-old mice (upper panels). Total mammary cells as well as frequencies of CD45^+^ and CD11b^+^F4/80^+^ cells are shown (lower panels). n = 4. Mann–Whitney U test. **(D)** Flow cytometry analysis of CD206, I-A/I-E, and CD86 expression in F4/80^+^ macrophages from mammary tissues of 30–35-week-old mice (upper panels). Frequencies and absolute numbers of CD206^+^ and I-A/I-E^+^ macrophages are shown (lower panels). n = 4. Mann–Whitney U test.

Whole mammary glands, excluding the draining lymph nodes, were digested with collagenase, and MECs (CD45^−^CD31^−^CD140a^−^) were analyzed by flow cytometry using antibodies against CD24 and CD49f. The total numbers of mammary gland cells per gland, MECs, CD49f^low^ luminal cells (integrin α6^low^), and CD49f^high^ basal cells (integrin α6^high^) were all significantly increased in IL-21isoTg mice compared with their levels in wild-type mice (**Figure 2A**).

Moreover, the mean fluorescence intensity (MFI) of CD49f was significantly elevated in IL-21isoTg mice relative to that in wild-type controls, suggesting phenotypic alterations in MECs (**Supplementary Figure 2**). Ki67 staining of each epithelial subset revealed a significantly higher proportion of Ki67-positive cells in IL-21isoTg mice (**Figure 2B**), which was consistent with the increased Ki67 staining observed in mammary tissue sections (**Supplementary Figure 1B**).

Next, we analyzed the CD45^+^ hematopoietic cells infiltrating the mammary glands. Although the overall proportion of CD45^+^ cells was reduced in IL-21isoTg mice due to the marked expansion of total mammary gland cells, the proportion of CD11b^+^F4/80^+^ macrophages within the CD45^+^ population was clearly increased (**Figure 2C**).

Further characterization of macrophage subsets using M1 markers (I-A/I-E or CD86) and M2 marker CD206 demonstrated a marked increase in both the number and proportion of M2 macrophages in IL-21isoTg mice. In contrast, the proportion of M1 macrophages was unchanged, although their absolute number was slightly increased in IL-21isoTg mice (**Figure 2D**). CD86-positive macrophages were rarely detected in mice of either genotype.

### 3.3 Upregulation of the OPN-encoding *Spp1* gene expression in MECs and stromal cells of IL-21isoTg mice

In humans, hormonal factors, such as estrogen, prolactin, and progesterone, are known to contribute to breast cancer development (47–49). We initially hypothesized that dysregulation of these hormones might underlie mammary tumor development in IL-21isoTg mice. However, serum levels of these hormones were comparable between the wild-type and IL-21isoTg mice. Notably, estrogen levels tended to be lower in the IL-21isoTg mice (**Supplementary Figure 3A**).

To further examine the role of estrogen, ovariectomy was performed in 8-week-old IL-21isoTg mice, and mammary tumor development was monitored. Mammary tumors developed in ovariectomized (OVX) IL-21isoTg mice at the same rate as in sham-operated IL-21isoTg controls (**Table 2**). These findings suggest that estrogen was unlikely essential for mammary tumor development in IL-21isoTg mice. The estrogen receptor in MECs tended to be expressed at higher levels in IL-21isoTg mice (**Supplementary Figures 3B**, left), suggesting the possibility of enhanced sensitivity to estrogen signaling. Further studies are required to clarify the role of estrogen signaling in tumor development using this model.

**Table 2:** Number of mice that developed tumors after OVX surgery.

Surgery	No tumor	Tumor
Sham	3	4
OVX	3	6

**Figure 3:**
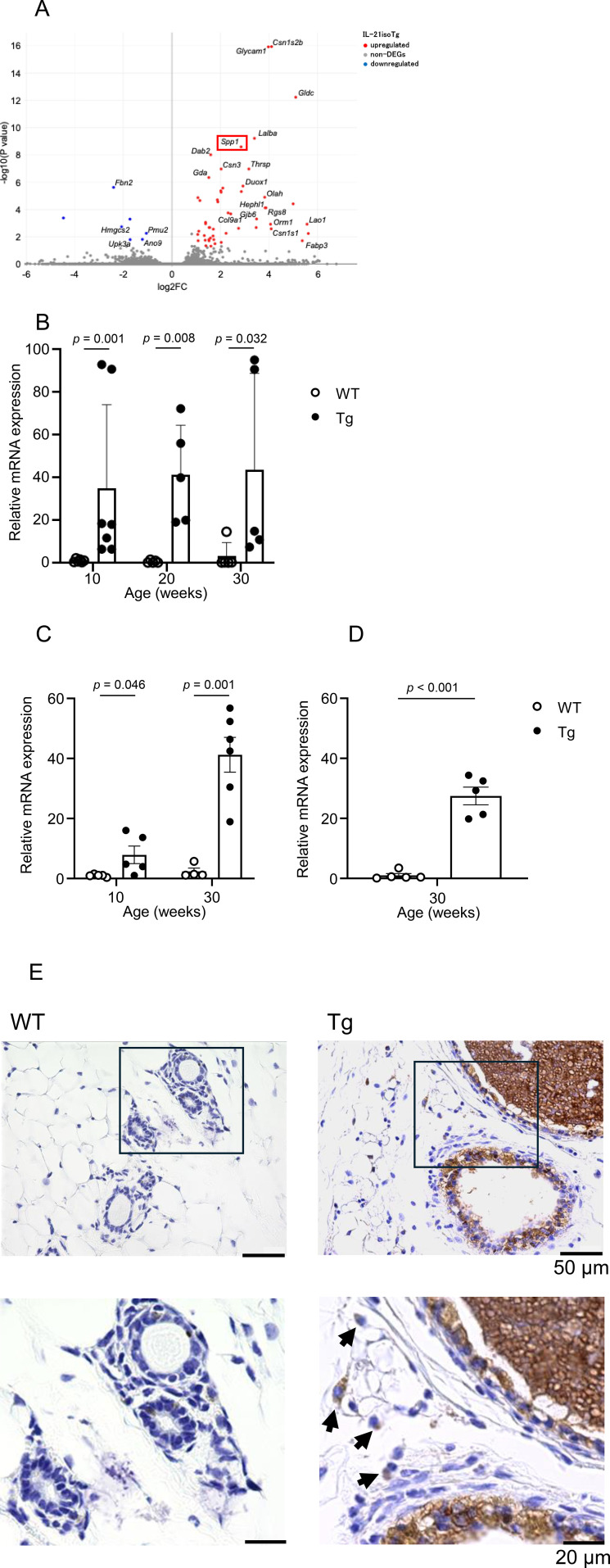
*Spp1* mRNA expression in MECs and stromal cells of IL-21isoTg mice. **(A)** Volcano plot of RNA-seq analysis of MECs from 10-week-old mice. Genes upregulated (red) and downregulated (blue) in IL-21isoTg mice in relation to their expression levels in wild-type mice are shown (n = 3). **(B)** RT-PCR analysis of *Spp1* expression in mammary tissues (n = 5–7). Mann–Whitney U test. **(C, D)** RT-PCR analysis of *Spp1* expression in MECs **(C)** and mammary stromal cells **(D)** (n = 4–6). Mann–Whitney U test. **(E)** Immunohistochemical analysis of OPN expression in mammary tissues from 35-week-old mice. Lower panels show higher magnification images of the indicated areas. Arrows in a lower panel indicate stromal cells expressing OPN.

To investigate early molecular alterations prior to tumor formation, MECs were isolated from 10-week-old mice and subjected to RNA sequencing analysis, followed by comparison with wild-type controls. IL-21isoTg MECs showed marked upregulation of genes associated with mammary gland development and lactation, including those encoding caseins and lactalbumin. In addition, the expression of *Spp1*, which encodes OPN, and *Gldc*, which encodes glycine decarboxylase (GLDC) were prominently activated (**Figure 3A**). In this study, we focused our analysis on OPN.

We quantified *Spp1* expression in whole mammary tissue, isolated MECs, and mammary stromal cells using real-time RT-PCR. *Spp1* expression was significantly elevated in the mammary glands of IL-21isoTg mice at 10, 20, and 30 weeks of age compared with that in wild-type mice (**Figure 3B**). Furthermore, sorted mammary epithelial and stromal cell fractions from IL-21isoTg mice both showed significantly increased *Spp1* expression relative to that in wild-type controls (**Figures 3C, D**).

Immunohistochemical analysis confirmed increased OPN protein expression in the mammary tissues of IL-21isoTg mice (**Figure 3E**). Compared with wild-type mice, OPN was strongly expressed in MECs, within dilated ducts, and in some stromal cells of IL-21isoTg mice, consistent with the RT-PCR findings (**Figures 3C, D**).

### 3.4 IL-21 induces OPN production in macrophages and B cells

**Figure 4:**
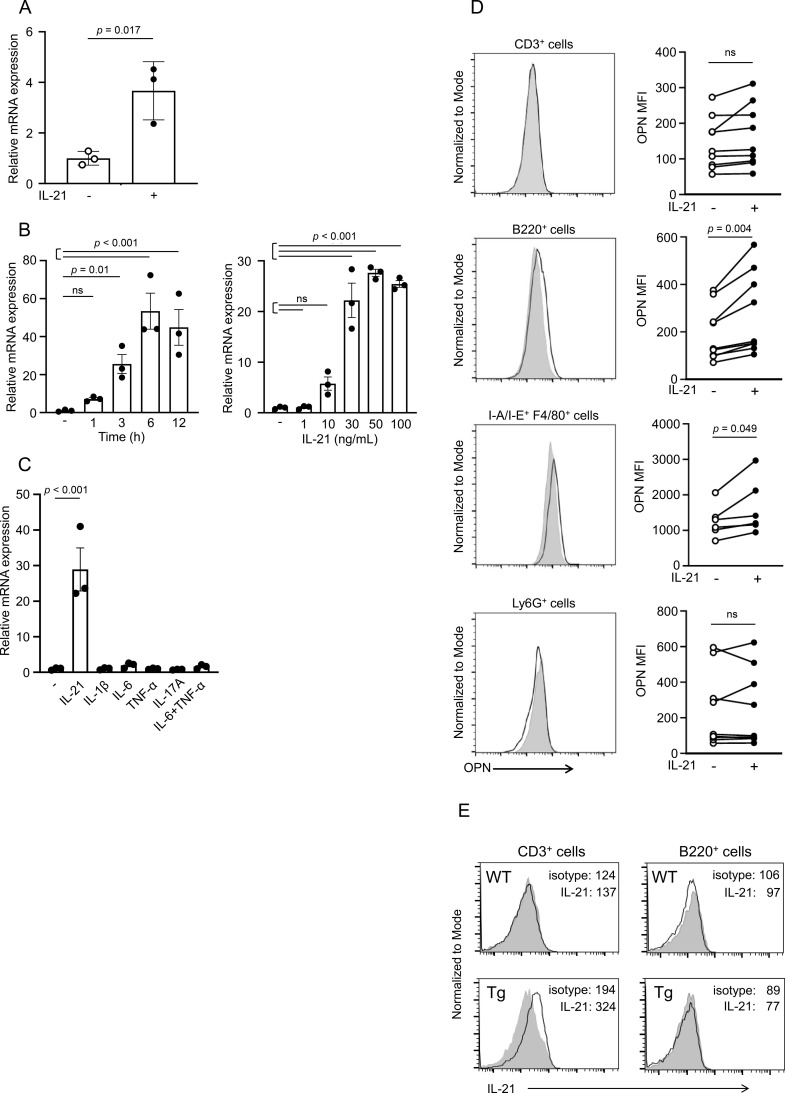
IL-21 induces OPN production in macrophages and B cells. **(A)** RT-PCR analysis of *Spp1* gene expression in mammary gland cells stimulated with 50 ng/mL IL-21 for 6 h. n = 3. Mann–Whitney U test. **(B)** RT-PCR analysis of *Spp1* gene expression in splenocytes stimulated with IL-21. Kinetic analysis following stimulation with 50 ng/mL IL-21 for up to 12 h (left) and dose–response analysis following 6 h stimulation (right). n = 3. Ordinary one-way ANOVA with Dunnett’s multiple comparisons test. **(C)** Splenocytes were stimulated with various cytokines for 6 h, and *Spp1* expression was analyzed by RT-PCR. Cytokine concentrations: IL-21 (50 ng/mL), IL-1β (50 ng/mL), IL-6 (50 ng/mL), TNF-α (100 ng/mL), IL-17 (100 ng/mL). n = 3. Ordinary one-way ANOVA with Dunnett’s multiple comparisons test. **(D)** Representative flow cytometry analysis of OPN expression in splenocytes stimulated with IL-21 (50 ng/mL) for 24 h (left). Quantification is shown (right). Paired two-tailed *t*-test. CD3^+^ cells, B220^+^ cells, and Ly6G^+^ cells (n = 9); I-A/I-E^+^ F4/80^+^ cells (n = 6). ns, not significant. **(E)** Flow cytometry analysis of IL-21 expression in CD3^+^ T cells and B220^+^ B cells among mammary stromal cells. The numbers shown in the histograms indicate the respective MFIs.

In IL-21isoTg mice, *Spp*1 expression was markedly elevated not only in MECs but also in mammary stromal cells (**Figure 3D**). To determine whether this increase in *Spp1* expression was induced by IL-21, mammary gland cells isolated from wild-type mice were stimulated *ex vivo* with IL-21. IL-21 significantly upregulated *Spp1* expression in mammary gland cells (**Figure 4A**). Because MECs do not express the IL-21 receptor, this activation of *Spp1* is presumed to be mainly mediated by stromal cells in the mammary gland. To further elucidate the mechanisms underlying IL-21-induced *Spp1* expression, we employed wild-type splenocytes as a surrogate for the immune cell populations infiltrating the mammary gland. When splenocytes were stimulated *ex vivo* with IL-21, *Spp1* expression was induced as early as 3 h after the stimulation in an IL-21 dose-dependent manner (**Figure 4B**).

In contrast, cytokines previously reported to induce *Spp1* expression, such as IL-1β, IL-6, TNF-α, and IL-17A, had minimal effects on *Spp*1 mRNA levels in splenocytes under our experimental conditions (**Figure 4C**) (50–53).

Flow cytometry analysis (**Supplementary Figure 4**) showed that IL-21 stimulation predominantly induced OPN production in macrophages and B cells (**Figure 4D**).

These findings suggested that in IL-21isoTg mice, stromal cells accumulating in the mammary glands, such as macrophages and B cells, may also produce OPN in response to IL-21iso stimulation. Indeed, IL-21iso-expressing CD3^+^ T cells were detected in the mammary tissue of IL-21isoTg mice (**Figure 4E**).

Collectively, these results indicated that IL-21iso produced by T cells localized within the mammary glands induced OPN production in stromal cells such as macrophages and B cells, thereby contributing to the establishment of a tumor-promoting microenvironment.

Furthermore, we analyzed peripheral blood mononuclear cells (PBMCs) isolated from human peripheral blood. Consistent with our findings in mice, IL-21 stimulation tended to increase OPN expression in B cells and monocytes (**Supplementary Figure 5**).

### 3.5 IL-21 indirectly activates *Spp1* expression in MECs via IL-6 and TNF-α

Next, we investigated the mechanism underlying upregulation of *Spp1* expression in the MECs of IL-21isoTg mice. Because IL-21 receptor expression was not detected in MECs, we hypothesized that cytokines other than IL-21 induce *Spp1* expression.

**Figure 5:**
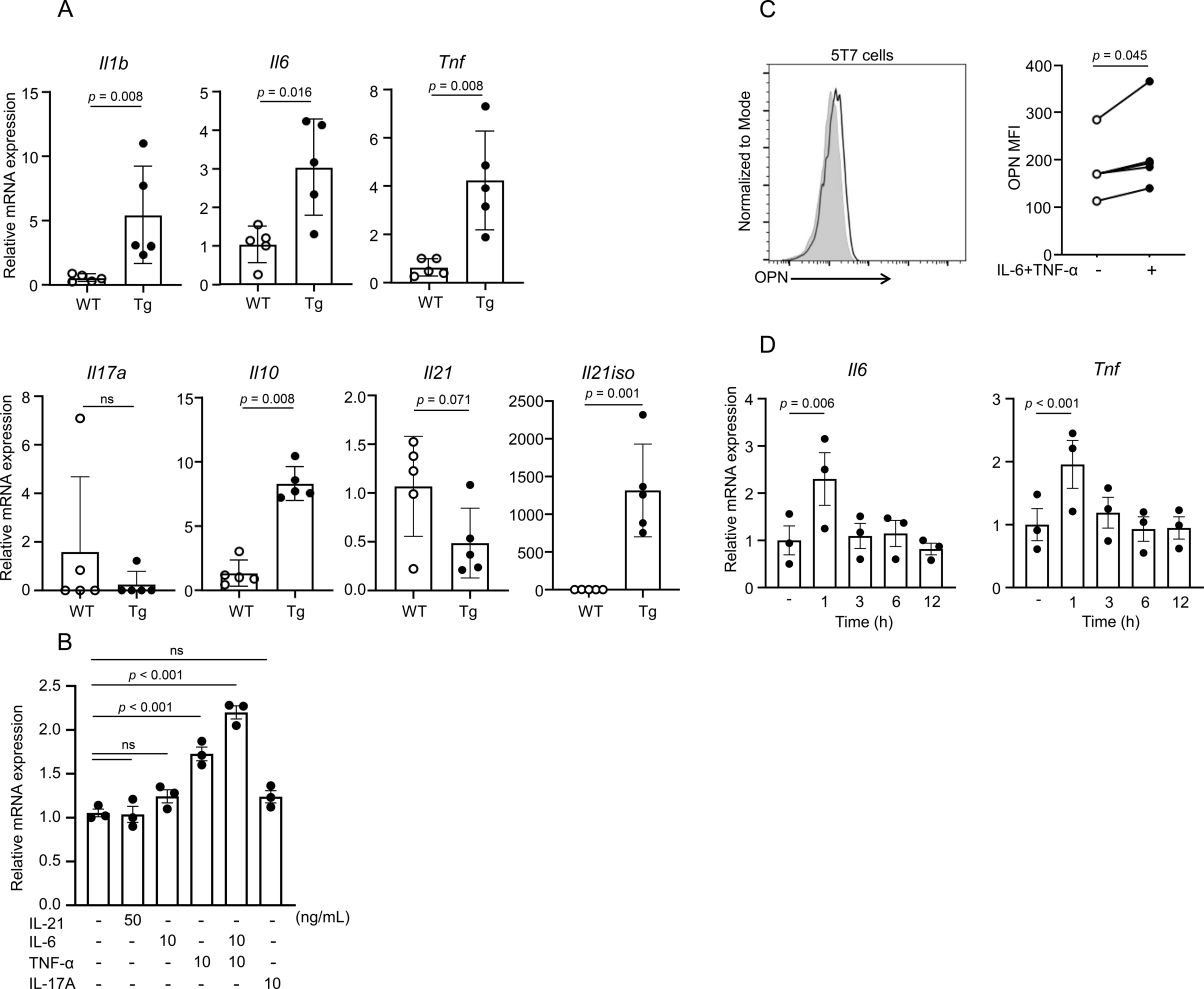
IL-21 activates *Spp1* expression in MECs via IL-6 and TNF-α. **(A)** RT-PCR analysis of cytokine gene expression in mammary tissues (n = 5). Mann–Whitney U test. **(B)** *Spp1* expression in 5T7 cells stimulated with various cytokines for 6 h, analyzed by RT-PCR (n = 3). Ordinary one-way ANOVA with Dunnett’s multiple comparisons test. **(C)** Representative flow cytometry analysis of OPN expression in 5T7 cells stimulated with 100 ng/mL IL-6 and 100 ng/mL TNF-α for 24 h (left). Quantification is shown (right). Paired two-tailed *t*-test, n = 5. **(D)** Activation of *Il6* and *Tnf* expression by IL-21. Splenocytes were stimulated with 50 ng/mL IL-21, and gene expression was analyzed by RT-PCR (n = 3). Ordinary one-way ANOVA with Dunnett’s multiple comparisons test.

Mammary tissues from 30-week-old IL-21isoTg mice had significantly higher expression levels of *Il1b*, *Il6*, *Tnf*, *Il10*, and *Il21iso* compared with those in mammary samples from wild-type mice (**Figure 5A**). Among these cytokines, IL-1β, IL-6, TNF-α, and IL-17A have previously been reported to enhance *Spp1* expression. Therefore, we stimulated cell line 5T7, one of the cell lines established from mammary gland tumors derived from IL-21isoTg mice, with each cytokine and IL-21 for 6 hours. These cell lines were positive for CD24, keratin 7, and EpCAM, as well as weakly positive for CD49f, indicating a luminal epithelial origin (**Supplementary Figure 6**). In addition, these cell lines were negative for estrogen and progesterone receptors but positive for ERBB2, which is consistent with a defined subtype of human mammary tumor cells (**Supplementary Figure 6**).

Stimulation with IL-6 and TNF-α significantly and additively enhanced *Spp1* expression in 5T7 cells (**Figure 5B**). Similar to primary MECs, 5T7 cells did not express the IL-21 receptor (data not shown) and stimulation with either IL-21 or IL-17A did not increase *Spp1* expression levels. Upregulation of OPN production by IL-6 and TNF-α in 5T7 cells was confirmed by flow cytometry (**Figure 5C**).

IL-21 stimulation promoted *Il6* and *Tnf* expression in splenocytes (**Figure 5D**). These results suggest that in IL-21isoTg mice, IL-21iso-producing T cells accumulated in the mammary gland induce IL-6 and TNF-α production, which in turn may activate *Spp1* expression in MECs.

### 3.6 Impaired antitumor immunity in IL-21isoTg mice

Given the increased OPN expression in the mammary tissues of IL-21isoTg mice and spontaneous development of mammary tumors, we next examined whether antitumor immunity was altered in this model.

**Figure 6:**
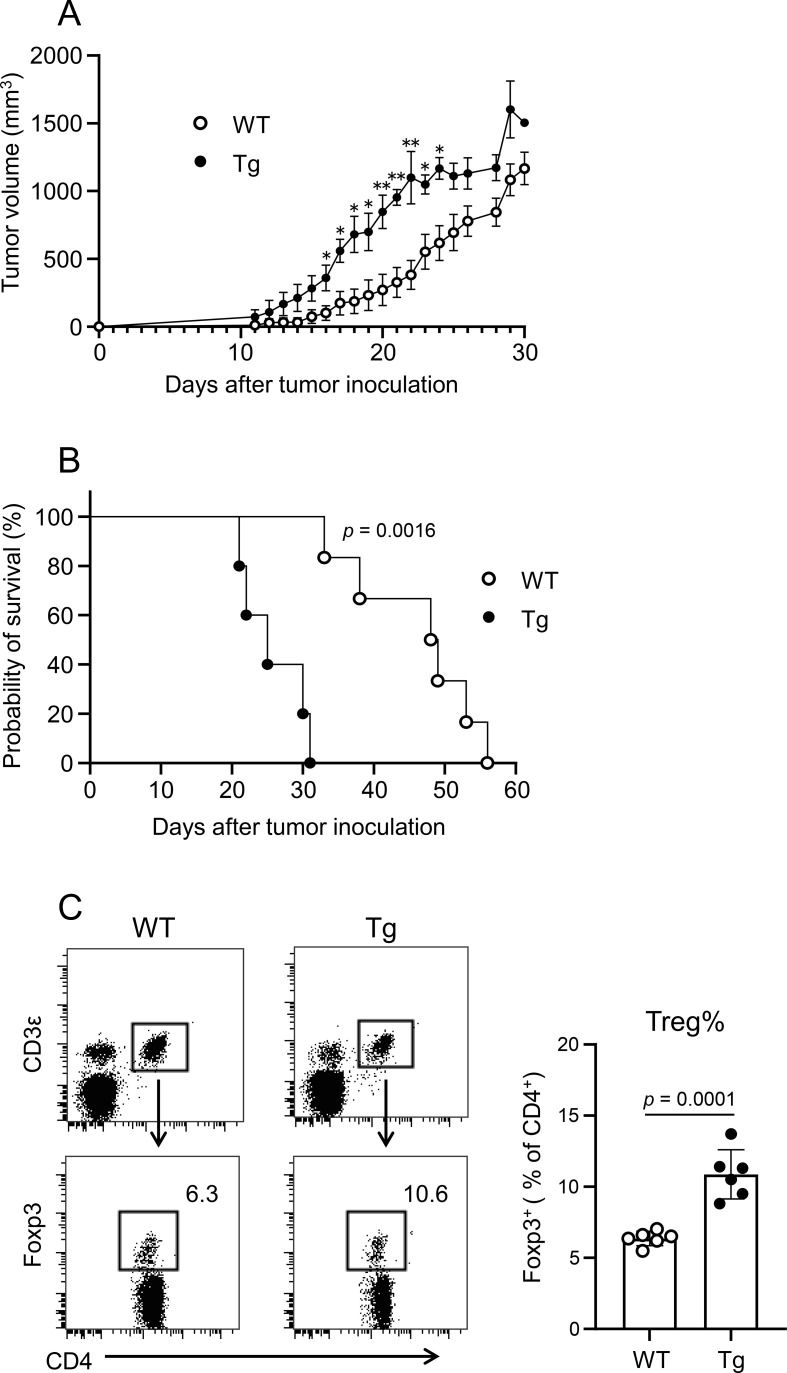
Impaired antitumor immunity in IL-21isoTg mice. **(A)** Growth of subcutaneously transplanted Renca cells in wild-type (n = 6) and IL-21isoTg mice (n = 5). Two-tailed *t*-test. *p < 0.05, **p < 0.01. **(B)** Survival of wild-type (n = 6) and IL-21isoTg mice (n = 5) after Renca cell transplantation. Statistical analysis was performed using the Gehan–Breslow–Wilcoxon test. **(C)** Flow cytometry analysis of regulatory T cells (Tregs) among peripheral blood CD4^+^ T cells. The percentage of Foxp3^+^ cells within CD4^+^CD3ϵ^+^ T cells in the peripheral blood lymphocyte population of 10-week-old mice (n = 6). Statistical analysis was performed using the Mann–Whitney U test.

Renca cells, a BALB/c-derived renal carcinoma cell line, were subcutaneously inoculated into mice, and tumor growth was monitored. Tumor progression was significantly accelerated in IL-21isoTg mice compared with that in wild-type controls (**Figure 6A**). Consistent with enhanced tumor growth, overall survival was reduced in IL-21isoTg mice (**Figure 6B**). Furthermore, in IL-21isoTg mice, the proportion of regulatory T cells (Tregs) among peripheral blood CD4^+^ T cells were significantly increased compared to wild-type mice (**Figure 6C**).

These results indicate that IL-21isoTg mice have impaired antitumor immunity from a relatively early age. This reduction in antitumor immunity may contribute to the development of mammary tumors in this model.

## 4 Discussion

Our previous studies demonstrated that IL-21isoTg mice exhibit increased susceptibility to DSS-induced colitis and colorectal tumorigenesis caused by combined treatment with DSS and azoxymethane (28, 54), suggesting that IL-21 functions as a pro-inflammatory cytokine. It has recently been reported that CD4^+^ T cells from elderly individuals produce higher levels of IL-21 compared to those from younger individuals (29). Furthermore, serum IL-21 levels are elevated in patients with Alzheimer’s disease and mild cognitive impairment—conditions that increase in prevalence with age—suggesting that IL-21 may be involved in the regulation of microglial function (30, 31).

To examine the long-term consequences of chronic IL-21 elevation, we monitored IL-21isoTg mice over an extended period. We found that more than 45% of IL-21isoTg mice spontaneously developed mammary tumors after 12 months of age. Notably, ductal dilation and other morphological abnormalities were observed as early as 14 weeks of age, suggesting that chronic IL-21 stimulation may contribute to the early mammary epithelial alterations that precede tumorigenesis.

In humans, hormonal factors such as estrogen, prolactin, and progesterone are known to be involved in the development of breast cancer (47–49). In IL-21isoTg mice, however, no increases in serum estrogen or prolactin levels were observed. Furthermore, ovariectomy did not affect the development of mammary tumors in these mice. These results suggest that the development of mammary tumors in the IL-21isoTg model involves mechanisms distinct from those typically observed in humans.

RNA-seq analysis of MECs from 10-week-old IL-21isoTg mice revealed a marked upregulation of *Spp1* expression, which was detected not only in MECs but also in stromal cells within the mammary gland. These alterations occurred prior to tumor development, strongly suggesting that they contributed to tumorigenesis.

In addition to *Spp1*, RNA-seq analysis revealed that *Gldc* expression was also significantly elevated in the mammary epithelium of IL-21isoTg mice. GLDC (glycine decarboxylase) is a key mitochondrial enzyme of the glycine cleavage system (GCS) that catalyzes the degradation of glycine to generate one-carbon units (55). Previous studies have demonstrated that GLDC is profoundly involved in tumor cell proliferation and tumorigenesis across various cancer types. For instance, in non-small cell lung cancer (NSCLC), GLDC is essential for the growth of tumor-initiating cells (TICs) and is sufficient to drive cellular transformation (56). Furthermore, aberrant GLDC expression has been observed in several malignancies, including MYCN-amplified neuroblastoma (57). Although the specific role of GLDC in breast cancer development remains largely unexplored, its marked upregulation in the IL-21isoTg model is a highly intriguing phenomenon. This suggests a potential link between IL-21 signaling and metabolic reprogramming during mammary tumorigenesis. Therefore, further investigation into the functional significance of GLDC in this context is warranted.

The expression and function of OPN in tumors have been extensively studied. High OPN expression in human cancers is widely associated with tumor progression, metastasis, and poor prognosis (37, 58, 59). OPN is also highly expressed in tumor-infiltrating macrophages and other myeloid-derived cells (60, 61). Furthermore, OPN-expressing macrophages have been shown to remodel the tumor microenvironment (TME) and suppress CD8^+^ T cell function in multiple cancer types (39, 41, 62, 63). Studies using OPN-deficient mice have also demonstrated that myeloid-derived OPN plays a more prominent role than tumor-derived OPN in mediating immunosuppression (67).

In the present study, we demonstrated that IL-21 is involved in OPN expression in both the mammary epithelium and mammary stromal cells. To investigate the clinical relevance, we examined publicly available dataset for a potential correlation between IL-21 and OPN in human breast cancer (64). Although we found no significant correlation between elevated OPN levels and increased IL-21 expression in the dataset, further detailed analysis remains necessary.

Our findings suggest that chronic IL-21 exposure in IL-21isoTg mice promotes OPN production in mammary stromal cells, potentially leading to immunosuppression. Consistent with this interpretation, IL-21isoTg mice exhibited a higher proportion of Tregs within the CD4^+^ population compared to wild-type mice. Furthermore, subcutaneous transplantation of Renca cells significantly accelerated tumor growth and reduced survival in IL-21isoTg mice, indicating impaired anti-tumor immunity.

This finding stands in contrast to the established role of IL-21 in enhancing CD8^+^ T cell function and preventing exhaustion (21–24). Due to its potent anti-tumor activity, IL-21 has been evaluated in various clinical trials for malignancies such as melanoma and renal cell carcinoma (65). For example, combination therapy with IL-21 and anti-EGFR antibodies for metastatic colorectal cancer resulted in stable disease (SD) in approximately 60% of patients (66). Recent studies have renewed interest in IL-21 as a promising therapeutic adjuvant, showing enhanced efficacy when combined with other immunotherapies, such as anti-HER2 antibodies or HER2-specific cytotoxic T lymphocytes (CTLs) (67–69).

Therefore, the immunosuppressive effects identified here in IL-21isoTg mice contradict the classical tumor-enhancing effects of IL-21. This discrepancy may be attributed to the pleiotropic nature of IL-21, whose biological impact appears highly context-dependent. Unlike the transient administration of IL-21 in clinical settings, long-term exposure—as modeled in IL-21isoTg mice—may induce sustained OPN expression in both immune cells and the mammary epithelium. This persistent induction could, directly or indirectly, shift the local environment from anti-tumorigenic to pro-tumorigenic, ultimately promoting mammary tumor development. The precise mechanisms by which the IL-21-OPN axis contributes to impaired immunosurveillance require further elucidation.

In summary, OPN expression was induced in both MECs and mammary stromal cells of IL-21isoTg mice even before tumor onset. While the direct functional relationship between elevated OPN and tumor progression in IL-21isoTg mice requires further study through specific functional assays, these results suggest that increased OPN expression contributes to the formation of a microenvironment that facilitates mammary tumorigenesis. These findings emphasize the importance of cautiously evaluating the potential adverse effects of IL-21 in clinical applications. Furthermore, as IL-21 production increases with age, it is crucial to investigate whether elevated IL-21 levels contribute to the increased risk of cancer in the elderly.

## Data Availability

RNA-Seq data have been deposited in the National Center for Biotechnology Gene Expression Omnibus (GEO) public database and are publicly available as of the date of publication under accession number “GSE331007”.

## Supplementary material

Supplementary Figure 1 **(A)** Anatomical distribution of tumor development in IL-21isoTg mice (n = 30). **(B)** Ki67 staining of non-tumor mammary tissues (upper panels). Quantification of Ki67-positive cells in five high-power fields (×200 magnification). Wild-type mice (open circles) and IL-21isoTg mice (closed circles). Statistical analysis was performed using two-way ANOVA followed by the Tukey’s multiple comparisons test (n = 5). **(C)** Whole-mount staining of mammary glands from 20-week-old mice showing low-magnification (left) and high-magnification (right) views.

Supplementary Figure 2 Comparison of the MFI of CD49f expression in luminal and basal cells from 16–19-week-old wild-type and IL-21isoTg mice. Mann–Whitney U test.

Supplementary Figure 3 **(A)** Serum estrogen and prolactin concentrations measured by ELISA (n = 5–9). Mann–Whitney U test. **(B)** RT-PCR analysis of estrogen receptor and prolactin receptor expression in MECs (n = 4–6). Mann–Whitney U test.

Supplementary Figure 4 Flow cytometry gating strategy of splenocytes. **(A)** Splenocytes were stained for B220 and CD3. B220^+^ B cells (red gate) and CD3^+^ T cells (green gate) are shown. B220^−^CD3^−^ cells were further gated and analyzed for Ly6G and Ly6C expression. Ly6G^+^Ly6C^+^ neutrophils are shown (blue gate). **(B)** CD45^+^ cells were analyzed for Gr-1 and CD11c expression. Gr-1^−^ cells were further analyzed for I-A/I-E and F4/80 expression. I-A/I-E^+^F4/80^+^ macrophages are shown (yellow gate).

Supplementary Figure 5 IL-21 induces OPN expression in human monocytes and B cells. This study was conducted in accordance with the Declaration of Helsinki and approved by the Ethics Committee of the Yamagata University Faculty of Medicine (approval no. 2024-44). Informed consent was obtained from all participants involved in the study. Peripheral blood (5 mL) were collected from 5 healthy volunteers and heparinized at 5 U/mL with low molecular-weight heparin (Mochida Pharmaceutical Co., Tokyo, Japan). The average age of the donors was 44.2 ± 11.8 years (female 2, male 3; range of age 32 – 56). PBMCs were isolated from the heparinized whole blood using Ficoll-Paque PLUS (Cat. No. 17-1440-02, GE healthcare, Uppsala, Sweden). Briefly, the blood was diluted 1:1 with RPMI 1640 and carefully layered over the Ficoll-Paque solution. The tubes were centrifuged at 400 × g for 30 min at room temperature (20−25 °C). The mononuclear cell layer at the interface was harvested and washed with the culture medium (RPMI 1640 containing 10% heat-inactivated fetal calf serum) by centrifugation at 300 × g for 5 min. Cell viability and counts were assessed by trypan blue exclusion. PBMCs (1 × 10^6^ cells/mL) were stimulated with human IL-21 (50 ng/mL; Cat. No. AF-200-21, PeproTech, Rocky Hill, NJ) for 24 h. The cell staining was performed as described in Materials and Methods section (Flow cytometry). Human TruStain FcX (Cat. No. 422302, BioLegend) was used for Fc blocking prior to antibody staining. The antibodies used in this study were as follows: Pacific Blue-conjugated mouse anti-human CD3 mAb (clone UCHT1, Cat. No. 300431, BioLegend), PE-conjugated mouse anti-human CD14 mAb (clone MϕP9, Cat. No. BD556821, BD Biosciences), FITC-conjugated mouse anti-human CD19 mAb (clone HIB19, Cat. No. 302205, BioLegend). **(A)** Gating strategy for the identification of human PBMC subsets. Doublets are excluded using FSC-H vs. FSC-W and SSC-H vs. SSC-W plots to gate on single cells. CD3^+^, CD14^+^, and CD19^+^ cells are identified as T cells, monocytes, and B cells, respectively. **(B)** OPN expression levels in each PBMC subset. PBMCs were cultured for 24 h in the presence or absence of IL-21. Representative overlay histograms (vehicle, gray; IL-21, colored) are shown in the upper panels. Summarized the MFI of OPN in CD3^+^, CD19^+^, and CD14^+^ cells are presented as paired dot plots (lower panels). Lines connect data points from the same individual (n = 5). Data are representative of two independent experiments. *P* values are calculated using a two-tailed paired Student’s t-test; ns, not significant.

Supplementary Figure 6 Characterization of mammary tumor-derived cell lines. Flow cytometry of CD24 and CD49f expression (upper panels). RT-PCR analysis of gene expression in MECs, ovary and tumor cell lines (5T7 and 2T10).
