# Correction: IL-21 isoform transgenic mice spontaneously develop mammary tumors: possible involvement of IL-21-induced osteopontin expression in tumorigenesis

**DOI:** 10.3389/fimmu.2026.1904594

**Published:** 2026-06-15

**Authors:** Risako Yamaguchi, Akemi Araki, Yuji Takeda, Shinichi Saitoh, Junji Yokozawa, Masahiro Yamamoto, Naing Ye Aung, Mitsuru Futakuchi, Satoru Nagase, Hironobu Asao

**Affiliations:** 1 Department of Immunology, Yamagata University Faculty of Medicine, Yamagata, Japan; 2 Department of Obstetrics and Gynecology, Yamagata University Faculty of Medicine, Yamagata, Japan; 3 Department of Tumor Pathology, Faculty of Life Sciences, Kumamoto University, Kumamoto, Japan; 4 Department of Pathology, Yamagata University Faculty of Medicine, Yamagata, Japan

**Keywords:** immunosuppression, interleukin-21, interleukin-21 isoform, mammary tumor, osteopontin

Author RY and AA was erroneously omitted as equal contributing author. The original version of this article has been updated.

