# Supplementary figures and images for: IL-21 isoform transgenic mice spontaneously develop mammary tumors: possible involvement of IL-21-induced osteopontin expression in tumorigenesis
Source: Front Immunol. 2026 Jun 1;17:1834095. doi: 10.3389/fimmu.2026.1834095 (PMC13312059; doi:10.3389/fimmu.2026.1834095)

Suppl. Fig. 1C

WT

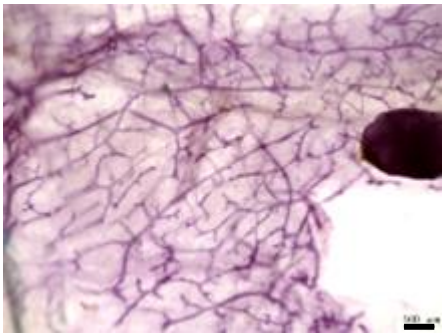

500  $\mu\text{m}$

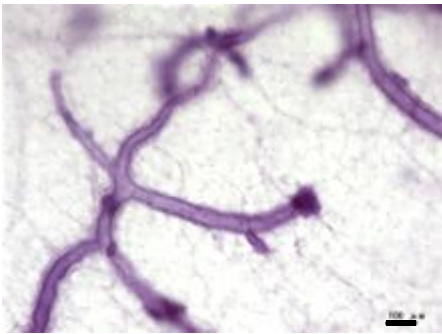

100  $\mu\text{m}$

Tg

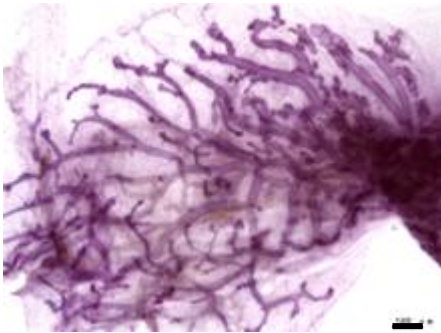

500  $\mu\text{m}$

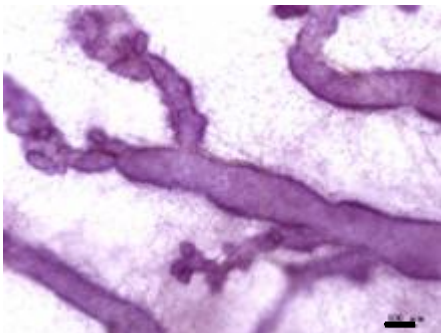

100  $\mu\text{m}$

Supplement: Supplementary Figure 1 — (A) Anatomical distribution of tumor development in IL-21isoTg mice (n = 30). (B) Ki67 staining of non-tumor mammary tissues (upper panels). Quantification of Ki67-positive cells in five high-power fields (×200 magnification). Wild-type mice (open circles) and IL-21isoTg mice (closed circles). Statistical analysis was performed using two-way ANOVA followed by the Tukey’s multiple comparisons test (n = 5). (C) Whole-mount staining of mammary glands from 20-week-old mice showing low-magnification (left) and high-magnification (right) views. [file Image1.pdf]

Suppl. Fig. 2

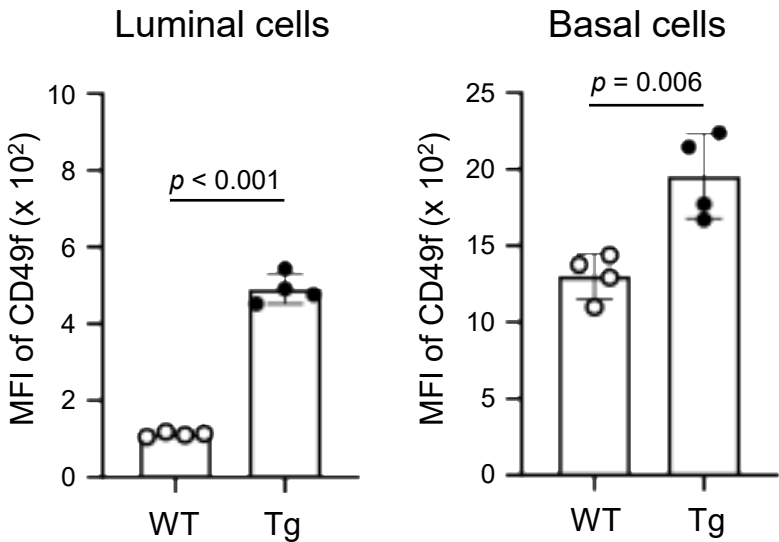

Supplement: Supplementary Figure 2 — Comparison of the MFI of CD49f expression in luminal and basal cells from 16–19-week-old wild-type and IL-21isoTg mice. Mann–Whitney U test. [file Image2.pdf]

Suppl. Fig. 3A

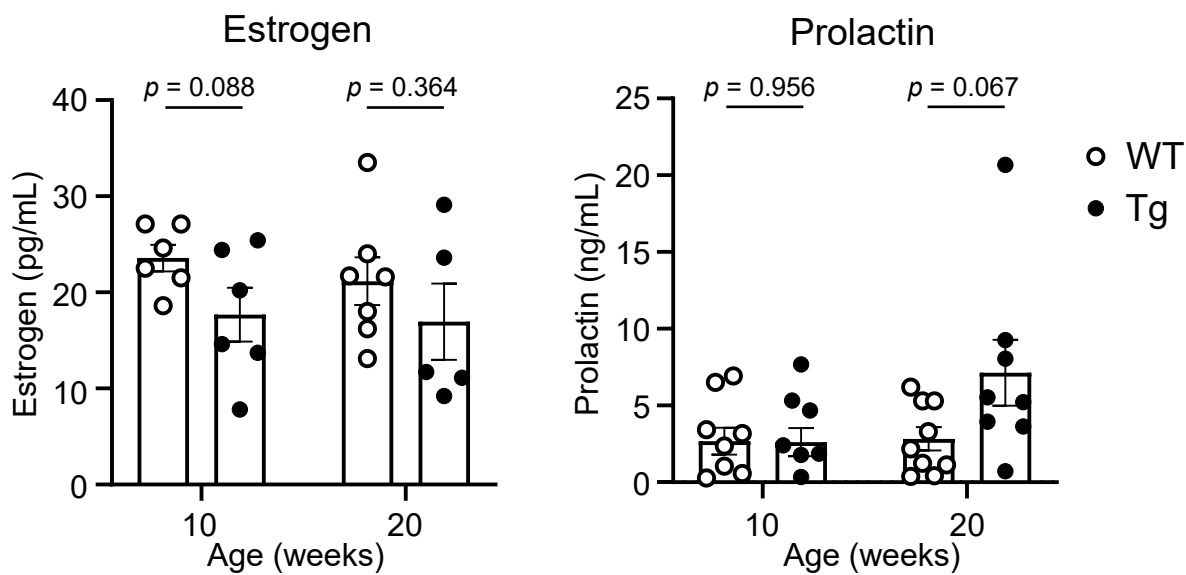

Suppl. Fig. 3B

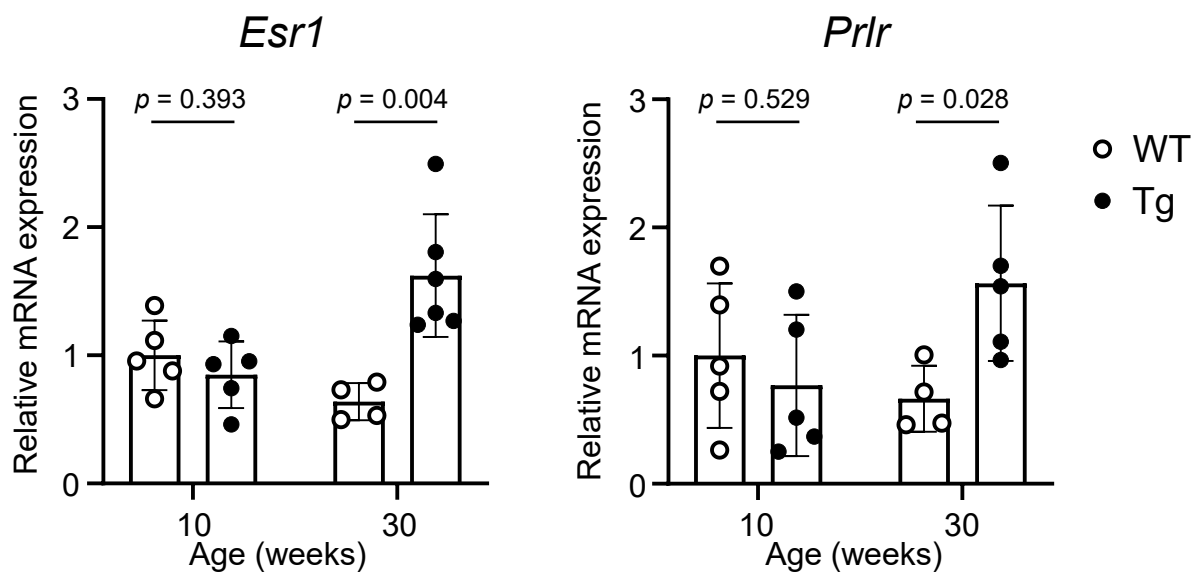

Supplement: Supplementary Figure 3 — (A) Serum estrogen and prolactin concentrations measured by ELISA (n = 5–9). Mann–Whitney U test. (B) RT-PCR analysis of estrogen receptor and prolactin receptor expression in MECs (n = 4–6). Mann–Whitney U test. [file Image3.pdf]

Suppl. Fig. 4

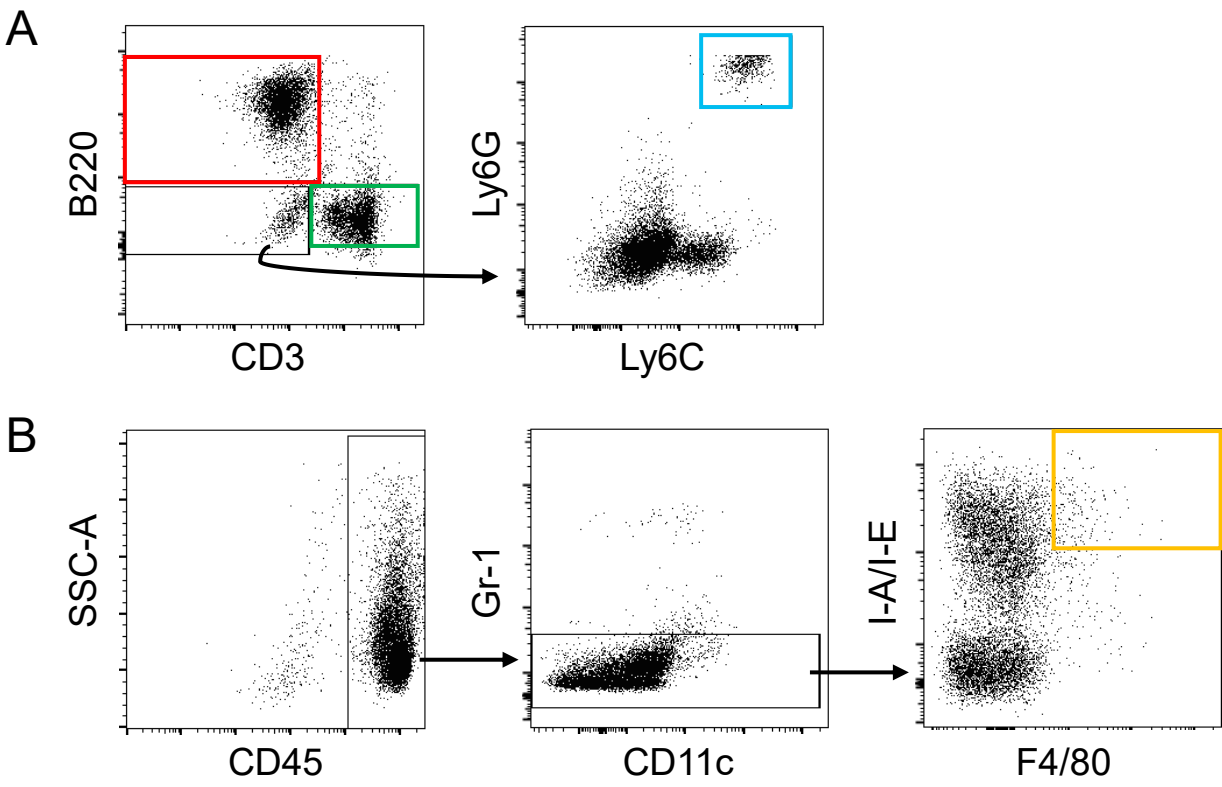

Supplement: Supplementary Figure 4 — Flow cytometry gating strategy of splenocytes. (A) Splenocytes were stained for B220 and CD3. B220+ B cells (red gate) and CD3+ T cells (green gate) are shown. B220−CD3− cells were further gated and analyzed for Ly6G and Ly6C expression. Ly6G+Ly6C+ neutrophils are shown (blue gate). (B) CD45+ cells were analyzed for Gr-1 and CD11c expression. Gr-1− cells were further analyzed for I-A/I-E and F4/80 expression. I-A/I-E+F4/80+ macrophages are shown (yellow gate). [file Image4.pdf]

A

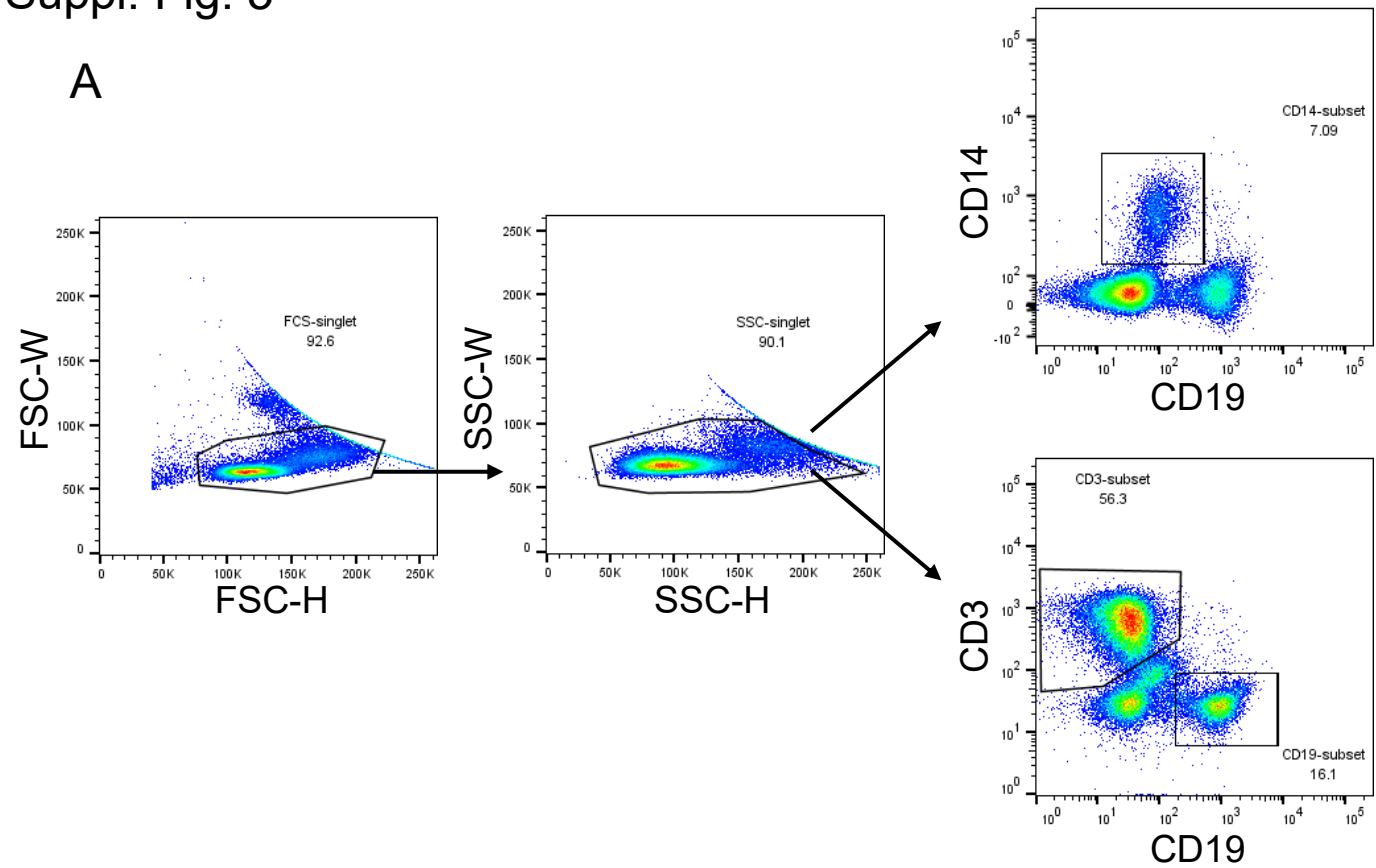

B

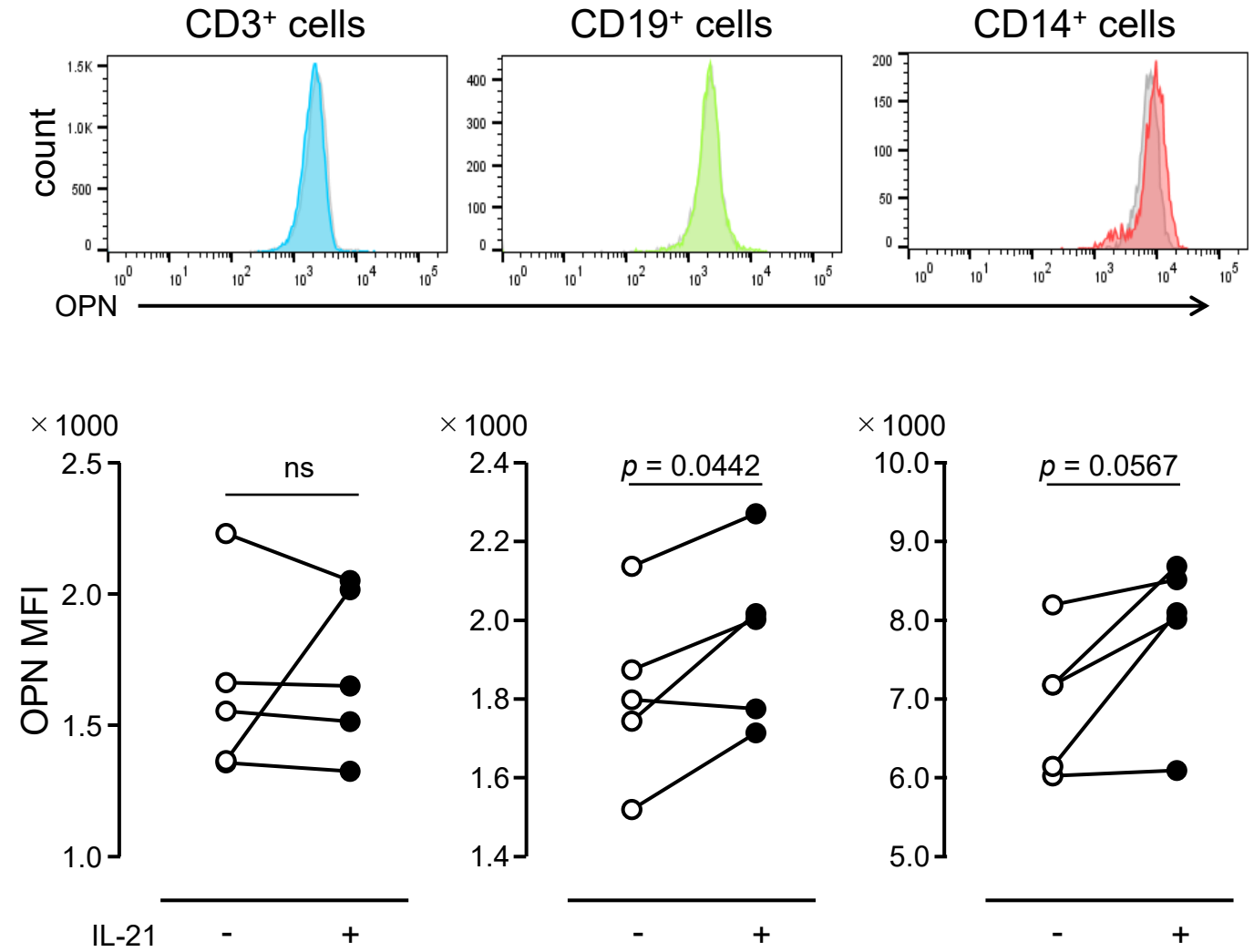

Supplement: Supplementary Figure 5 — IL-21 induces OPN expression in human monocytes and B cells. This study was conducted in accordance with the Declaration of Helsinki and approved by the Ethics Committee of the Yamagata University Faculty of Medicine (approval no. 2024-44). Informed consent was obtained from all participants involved in the study. Peripheral blood (5 mL) were collected from 5 healthy volunteers and heparinized at 5 U/mL with low molecular-weight heparin (Mochida Pharmaceutical Co., Tokyo, Japan). The average age of the donors was 44.2 ± 11.8 years (female 2, male 3; range of age 32 – 56). PBMCs were isolated from the heparinized whole blood using Ficoll-Paque PLUS (Cat. No. 17-1440-02, GE healthcare, Uppsala, Sweden). Briefly, the blood was diluted 1:1 with RPMI 1640 and carefully layered over the Ficoll-Paque solution. The tubes were centrifuged at 400 × g for 30 min at room temperature (20−25 °C). The mononuclear cell layer at the interface was harvested and washed with the culture medium (RPMI 1640 containing 10% heat-inactivated fetal calf serum) by centrifugation at 300 × g for 5 min. Cell viability and counts were assessed by trypan blue exclusion. PBMCs (1 × 10^6^ cells/mL) were stimulated with human IL-21 (50 ng/mL; Cat. No. AF-200-21, PeproTech, Rocky Hill, NJ) for 24 h. The cell staining was performed as described in Materials and Methods section (Flow cytometry). Human TruStain FcX (Cat. No. 422302, BioLegend) was used for Fc blocking prior to antibody staining. The antibodies used in this study were as follows: Pacific Blue-conjugated mouse anti-human CD3 mAb (clone UCHT1, Cat. No. 300431, BioLegend), PE-conjugated mouse anti-human CD14 mAb (clone MϕP9, Cat. No. BD556821, BD Biosciences), FITC-conjugated mouse anti-human CD19 mAb (clone HIB19, Cat. No. 302205, BioLegend). (A) Gating strategy for the identification of human PBMC subsets. Doublets are excluded using FSC-H vs. FSC-W and SSC-H vs. SSC-W plots to gate on single cells. CD3+, CD14+, and CD19+ cells are identified [file Image5.pdf]

Suppl. Fig. 6

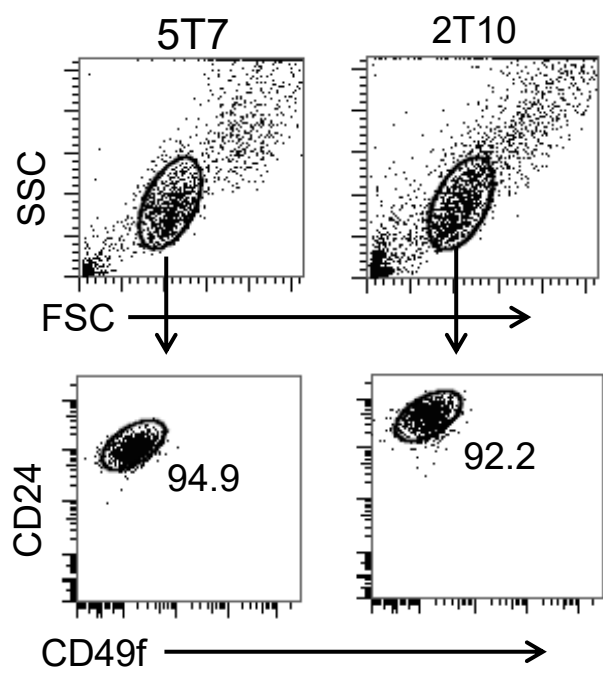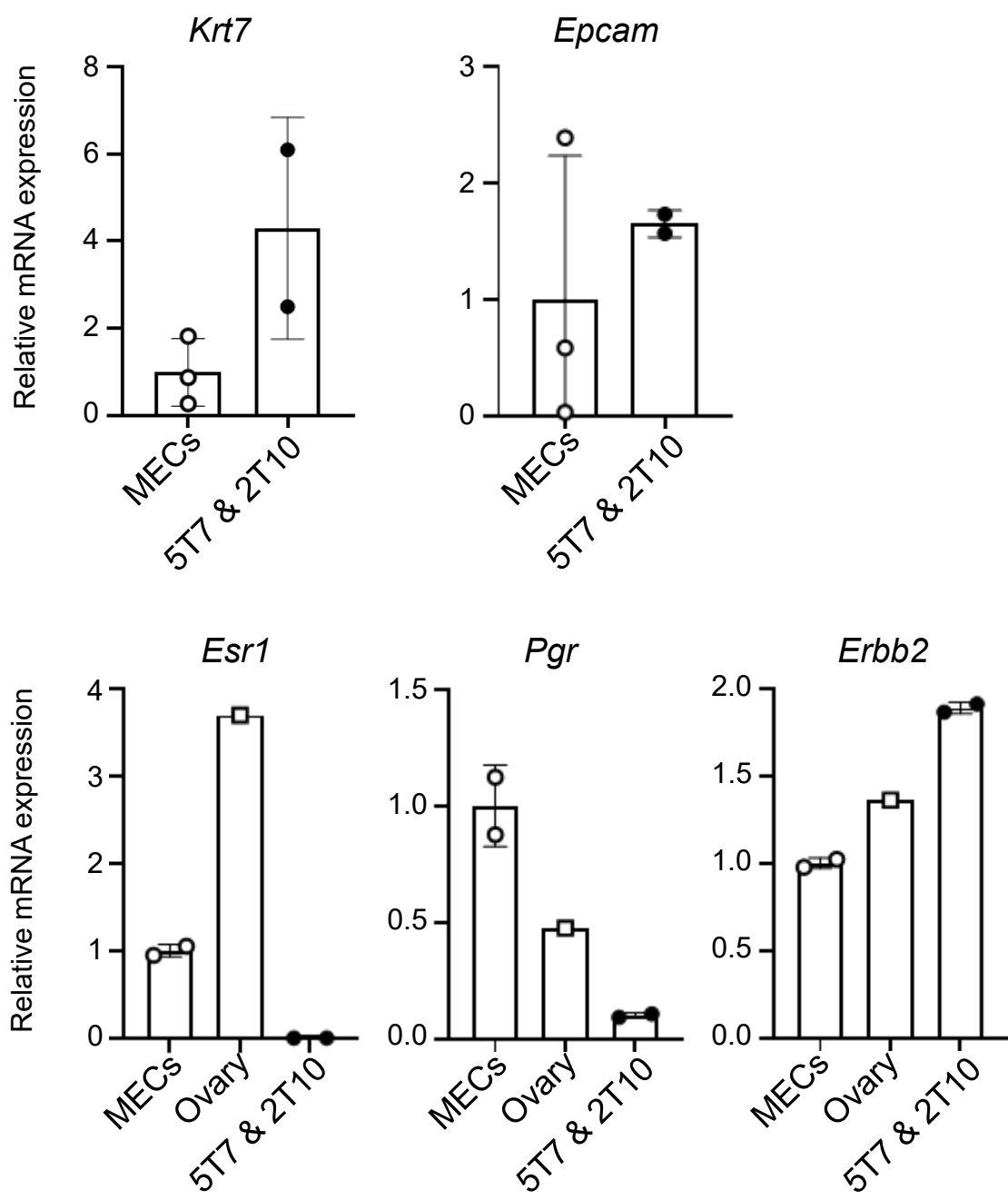

Supplement: Supplementary Figure 6 — Characterization of mammary tumor-derived cell lines. Flow cytometry of CD24 and CD49f expression (upper panels). RT-PCR analysis of gene expression in MECs, ovary and tumor cell lines (5T7 and 2T10). [file Image6.pdf]

Suppl. Fig. 1A

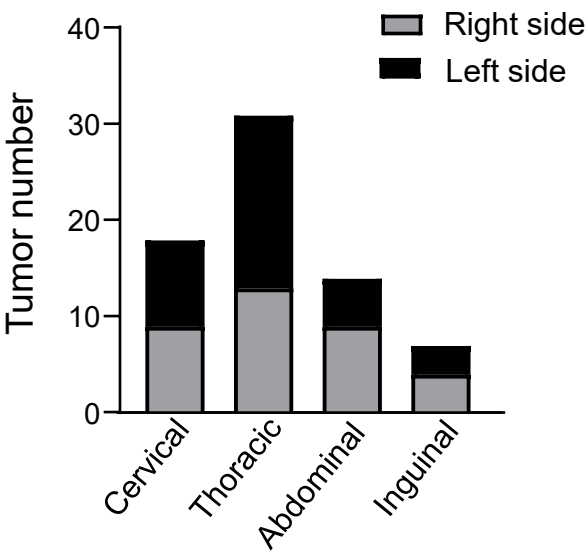

Suppl. Fig. 1B

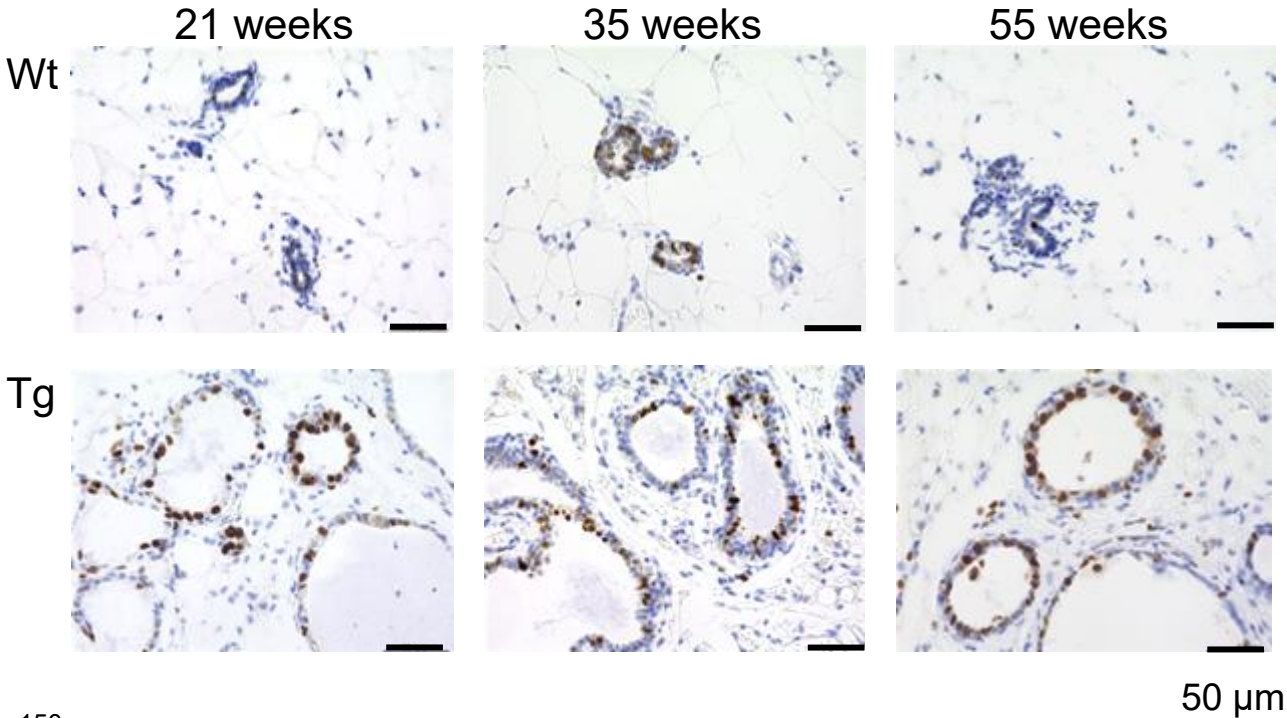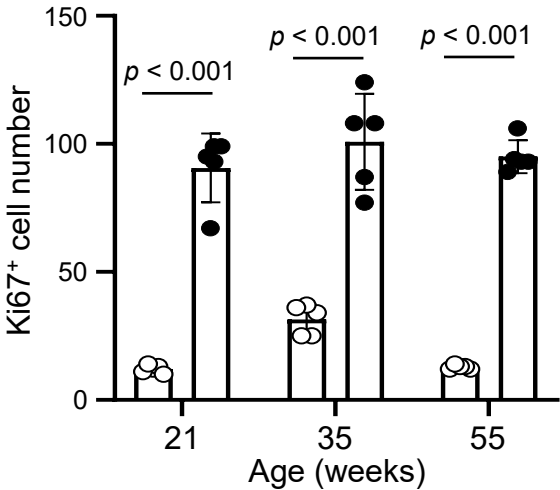

Supplement: Supplementary file 7 [file Image7.pdf]
